# Mechanical Performance of Advanced Composite Materials and Structures

**DOI:** 10.3390/ma17102172

**Published:** 2024-05-07

**Authors:** Yin Fan

**Affiliations:** School of Aeronautics and Astronautics, Shanghai Jiao Tong University, Shanghai 200240, China; yfan1987@sjtu.edu.cn

## 1. Introduction and Summary

In the realm of material science and engineering, the pursuit of lighter, stronger, and more durable materials has been an enduring quest. In recent decades, advanced composite materials have emerged as frontrunners in revolutionizing various industries, from aerospace and automotive to construction and sports equipment. Undoubtedly, mechanical performance is one of the most important attributes of composite materials and structures. On the one hand, the mechanical properties are expected to be fully explored and utilized to meet engineering requirements. In addition, improved process levels for existing composite materials and new fabrication methods for novel composite materials are other effective approaches to enhance their mechanical behaviors, leading to an industrial revolution.

This Special Issue on “Mechanical Performance of Advanced Composite Materials and Structures” is devoted to the most relevant studies related to advanced reinforced composites and their mechanical performances. The Guest Editor of the Special Issue, Dr. Yin Fan, has proposed to collate submissions on the following topics according to different types of materials: carbon fiber-reinforced polymer, metal matrix composite, nano reinforcement, auxetic lattice, and other composites. The suggested application areas include aerospace, civil engineering, sensors, and medical equipment. Fourteen research articles from China, America, Germany, Italy, Portugal, Poland, Austria, and Saudi Arabia were selected, peer-reviewed, and accepted for publication. These articles cover all aspects of the mechanical performance study of composite materials, such as numerical modeling, lay-up design, detection, mechanical tests, and fabrication. The range of research scales varies from nanometers to meters.

## 2. Contributions

### 2.1. Carbon Fiber-Reinforced Polymer

As is known, carbon fiber-reinforced polymer (CFRP), which is lightweight but high in strength, has been widely applied in aerospace engineering. To take full advantage of the potential of CFRPs, accurate and precise mechanical models are required in the design stage. In the paper entitled “Crash Performance of Inward-Inverting Composite Tubes Filled with Foam: Experimentation and Simulation”, Yu et al. [1] proposed a novel finite element model (FEM) adapted for shock absorber with an inward-inverting composite foam-filled tube to assess crashworthiness. The results revealed that the mean crush force from the simulation aligned well with the experimental data, with errors of the specific energy absorption being less than 7%. This indicates that the method is appropriate for the crush simulation of the proposed shock absorber.

CFRP joints have attracted the attention of engineers as they generally play an important role in aircraft structures. Two papers entitled “Study of Hybrid Composite Joints with Thin-Ply-Reinforced Adherends” and “Tests and Numerical Study of Single-Lap Thermoplastic Composite Joints Bolted by Countersunk” by Ramezani et al. [2] and Zhang et al. [3], respectively, explored the performance of CFRP joints using different methods of testing and FEM. However, the joint type was different: the first focused on bonded joints, whereas the second examined bolted joints. Both studies successfully established effective FEMs to simulate the damage propagation process of CFRP joints and obtained valuable results for practical engineering applications.

The designing of the stacking sequence of CFRP composite laminates inspired by nature may improve their desired mechanical properties. Yu et al. [4] investigated helicoidal laminates inspired by naturally occurring impact-resistant micro-structures in a research article entitled “Increasing the Compressive Strength of Helicoidal Laminates after Low-Velocity Impact upon Mixing with 0° Orientation Plies and Its Analysis”. In this study, they employed both numerical and experimental methods and found that laminates with pitch angles of 10° and 20° exhibited higher compression-after-impact (CAI) strengths, whereas using 0° layer could lead to large indentation areas. Hence, the authors concluded that the key parameters for CAI strength are pitch angles and the arrangement of 0° plies. Another paper on helicoidal CFRP laminate, titled “Optimization Design and Nonlinear Bending of Bio-Inspired Helicoidal Composite Laminated Plates” by Lu et al. [5], focused on the bending characteristics of helicoidal CFRP laminates and utilized FEM as an analytical tool. A parametric study revealed that single-form and combination-form helicoidal layups with lower rotation angles could decrease bending deflection under the boundary condition of four fixed edges.

Designing CFRP laminate layups can not only improve mechanical properties but also achieve abnormal auxeticity. Wang [6] contributed a paper entitled “Auxetic Composite Laminates with Through-Thickness Negative Poisson’s Ratio for Mitigating Low Velocity Impact Damage: A Numerical Study”, which proposed layups of CFRP composite laminates that produce negative Poisson’s ratios in the thickness direction. To directly exbibit the auxetic effect on the low-velocity impact of CFRP laminates, the author also identified two layups of non-auxetic CFRP counterpart laminates whose elastic moduli in three directions were very close to those of the auxetic laminate. Numerical comparison revealed that a negative Poisson’s ratio significantly influences the global impact response and damage behavior. Auxetic laminates with a through-thickness negative Poisson’s ratio consistently demonstrated higher impact forces, shorter impact times, reduced maximum displacements, and lower dissipated energies at elevated impact energy levels.

The use of CFRP is also garnering attention in medical apparatus and instruments due to its safety and comfort. Golewski et al. [7] conducted a study entitled “Composite Medical Tabletops Made of CFRP with Different Cross-Sections: Numerical Analysis and Laboratory Testing”, which was the first attempt to design CFRP medical tabletops. Numerical simulations and laboratory testing methods were used in the study for design and verification purposes, respectively. Four variants of CFRP medical tabletops were presented in the study, as well as three types of ergonomic tops. This work provides a reference for both researchers and designers involved in the medical application of CFRP.

Although most aerospace-grade CFRPs are made using thermoset polymers, thermoplastic polymers, such as PEEK (polyether ether ketone), are being considered as better substitutes due to their high mechanical, thermal, and chemical performance. Hümbert et al. [8] investigated the effect of processing parameters on in situ bonding between continuous fiber-reinforced PEEK laminates and short fiber-reinforced PEEK 3D printing during overprinting in a study entitled “Mechanical Characterisation of Bond Formation during Overprinting of PEEK Laminates”. The study found that the bonding strength primarily depended on temperature, with a maximum value reaching up to 15 MPa. The study results can guide future processes and the upscaling of the overprinting process using PEEK.

### 2.2. Metal Matrix Composite

Compared to CFRPs, metal matrix composites (MMCs) can serve at higher temperatures. Petrus et al. [9] proposed a novel alumina matrix composite reinforced with MAX phases (titanium aluminum carbide Ti_3_AlC_2_) in the paper entitled “Novel Alumina Matrix Composites Reinforced with MAX Phases—Microstructure Analysis and Mechanical Properties”. The authors emphasized that spark plasma sintering technology effectively reduces the decomposition of the used MAX phase. The application of MAX phases as a reinforcing phase in the alumina allows one to obtain sinters with high hardness, similar to the hardness of pure sinter while improving the fracture toughness. The composites demonstrated almost 20% higher fracture toughness while maintaining high hardness.

Currently, high-entropy alloys (HEAs) have attracted the attention of the research community owing to their excellent mechanical, wear, thermal, fatigue, and surface properties. Sivasankaran et al. [10] studied the influence of Al_2_O_3_ in CrFeCuMnNi high-entropy alloy matrix composites (HEMCs) on their microstructure, phase changes, and mechanical and wear performances in the paper “Effect of Al_2_O_3_ (x = 0, 1, 2, and 3 vol.%) in CrFeCuMnNi-x High-Entropy Alloy Matrix Composites on Their Microstructure and Mechanical and Wear Performance”. HEMCs were synthesized via mechanical alloying (MA) followed by hot compaction (550 °C at 550 MPa), medium frequency sintering (1200 °C), and hot forging (1000 °C at 50 MPa). The study found that the hardness and mechanical strength gradually increased, and the VHS and UCS of the CrFeCuMnNi-3 vol.% Al_2_O_3_ HEAMC sample were around 1.21 times higher than those of the unreinforced CrFeCuMnNi HEA matrix, correlating with the increase in the Al_2_O_3_ content.

### 2.3. Nano Reinforcement

Carbon nanotubes (CNTs), as one of the ideal potential fillers for nanocomposites [11], exhibit superior excellent mechanical properties compared to carbon fiber. In the paper “Chirality-Dependent and Intrinsic Auxeticity for Single-Walled Carbon Nanotubes”, Zhang et al. [12] discovered the auxetic nature of CNTs. They employed the molecular dynamics (MD) method to analyze the effect of chiral indices on the mechanical behavior, especially Poisson’s ratio, of CNTs. It was found that various chiral CNTs exhibited different critical strains, at which the incremental Poisson’s ratio could shift from negative from positive. Their study showed that the auxeticity of CNTs was mainly determined by the stretch of C-C bonds, and the critical strain was achieved when the negative contribution of bond stretch to Poisson’s ratio counteracted the positive contribution of angle variation. In their subsequent work, they proposed the atomic structures of auxetic nanocomposites [11] and verified their negative Poisson’s ratios using MD methods. Moreover, the mechanical performance of auxetic nanocomposites greatly surpassed that of existing man-made reentrant auxetic materials [13,14]. Thus, auxetic nanomaterials hold promise for future industrial applications.

### 2.4. Auxetic Lattice

Man-made auxetic materials exhibit densification behavior under compression or impact, making them suitable as cores in sandwiches with composite face sheets. In their article entitled “Crashworthiness of Additively Manufactured Auxetic Lattices: Repeated Impacts and Penetration Resistance”, Franzosi et al. [15] first selected and tested representative specimens, and then proceeded with an experimental and numerical study of repeated impact behavior and penetration resistance. Finally, they proposed a new design of a metallic auxetic absorber optimized for additive manufacturing and aimed at high-performance crash applications. Their work successfully demonstrated the potentiality of additively manufactured auxetic cellular structures for impact absorption, marking an advancement over previous literature in terms of empirical demonstrations of repeated impacts and penetration behavior.

### 2.5. Other Composites

In a paper entitled “Filament-Reinforced 3D Printing of Clay”, Jauk et al. [16] developed a method for filament-reinforced 3D printing of clay, along with relevant supporting hardware and software components. Mechanical tests showed that filament-reinforced 3D printing of clay is a viable solution that increases the tensile strength of unfired clay in both wet and dry states. Furthermore, when comparing unfired clay test specimens without and with filament reinforcement during printing, an increase of approximately 15% in tensile strength values along the axis of extrusion was observed.

Peng et al. [17] contributed a research article entitled “Assessing Effects of van der Waals Corrections on Elasticity of Mg_3_Bi_2_−_x_Sb_x_ in DFT Calculations”. They conducted a systematic investigation into the mechanical properties of the room-temperature thermoelectric material Mg_3_Bi_2_−_x_Sb_x_ (0 ≤ x ≤ 2) through first-principles calculations within the framework of density functional theory. The Perdew–Burke–Ernzerhof (PBE) exchange–correlation functional was used in calculations, and the mechanical properties, such as elastic constant and bulk modulus, were generally found to be within a range of 10%, except for a few outliers.

## 3. Outlook

This Special Issue of *Materials* was well supported by numerous submissions, and the final publication consists of 14 high-quality peer-reviewed articles. As the Guest Editor, I appreciate every author’s contribution to this Special Issue. It is anticipated that due to this success, a new Special Issue (“Mechanical Performance of Advanced Composite Materials and Structures (Second Edition)”; website: https://www.mdpi.com/journal/materials/special_issues/Q464B4HKW8, accessed on 22 November 2023) will be commissioned as a follow-up to accept global contributions.

## References

[1] Yu P., Yu Z., Zhou X., Xu W. (2023). Crash Performance of Inward-Inverting Composite Tubes Filled with Foam: Experimentation and Simulation. Materials.

[2] Ramezani F., Carbas R.J.C., Marques E.A.S., da Silva L.F.M. (2023). Study of Hybrid Composite Joints with Thin-Ply-Reinforced Adherends. Materials.

[3] Zhang J., Chen X., Tian A., Fan Y. (2022). Tests and Numerical Study of Single-Lap Thermoplastic Composite Joints Bolted by Countersunk. Materials.

[4] Yu Z., Du X., Liu R., Xie Q., Zhang X., Zhu Q. (2023). Increasing the Compressive Strength of Helicoidal Laminates after Low-Velocity Impact upon Mixing with 0° Orientation Plies and Its Analysis. Materials.

[5] Lu T., Shen H.-S., Wang H., Chen X., Feng M. (2023). Optimization Design and Nonlinear Bending of Bio-Inspired Helicoidal Composite Laminated Plates. Materials.

[6] Wang Y. (2022). Auxetic Composite Laminates with Through-Thickness Negative Poisson’s Ratio for Mitigating Low Velocity Impact Damage: A Numerical Study. Materials.

[7] Golewski P., Pietras D., Sadowski T., Wit-Rusiecki A.M. (2023). Composite Medical Tabletops Made of CFRP with Different Cross-Sections: Numerical Analysis and Laboratory Testing. Materials.

[8] Hümbert S., Atzler F., Voggenreiter H. (2024). Mechanical Characterisation of Bond Formation during Overprinting of PEEK Laminates. Materials.

[9] Petrus M., Wozniak J., Cygan T., Pawlak W., Olszyna A. (2022). Novel Alumina Matrix Composites Reinforced with MAX Phases—Microstructure Analysis and Mechanical Properties. Materials.

[10] Sivasankaran S., Ammar H.R., Sherif E.-S.M., Alaboodi A.S., Mekky A.-b.H. (2023). Effect of Al_2_O_3_ (x = 0, 1, 2, and 3 vol.%) in CrFeCuMnNi-x High-Entropy Alloy Matrix Composites on Their Microstructure and Mechanical and Wear Performance. Materials.

[11] Zhang H.-N., Fan Y., Shen H.-S. (2023). Prediction of Temperature-Dependent Mechanical Properties for SWCNT/Cu Nanocomposite Metamaterials: A Molecular Dynamics Study. Nanomaterials.

[12] Zhang H.-N., Fan Y., Shen H.-S. (2022). Chirality-Dependent and Intrinsic Auxeticity for Single-Walled Carbon Nanotubes. Materials.

[13] Fan Y., Xiang Y., Shen H.-S. (2020). Temperature-Dependent Mechanical Properties of Graphene/Cu Nanocomposites with In-Plane Negative Poisson’s Ratios. Research.

[14] Fan Y., Shen H.-S. (2022). Non-symmetric stiffness of origami-graphene metamaterial plates. Compos. Struct..

[15] Franzosi P., Colamartino I., Giustina A., Anghileri M., Boniardi M. (2024). Crashworthiness of Additively Manufactured Auxetic Lattices: Repeated Impacts and Penetration Resistance. Materials.

[16] Jauk J., Gosch L., Vašatko H., Königsberger M., Schlusche J., Stavric M. (2023). Filament-Reinforced 3D Printing of Clay. Materials.

[17] Peng Q., Ma X., Yang X., Zhao S., Yuan X., Chen X. (2023). Assessing Effects of van der Waals Corrections on Elasticity of Mg_3_Bi_2_−_x_Sb_x_ in DFT Calculations. Materials.

